# The glucagon-like peptide-1 receptor as a potential treatment target in alcohol use disorder: evidence from human genetic association studies and a mouse model of alcohol dependence

**DOI:** 10.1038/tp.2015.68

**Published:** 2015-06-16

**Authors:** P Suchankova, J Yan, M L Schwandt, B L Stangl, E C Caparelli, R Momenan, E Jerlhag, J A Engel, C A Hodgkinson, M Egli, M F Lopez, H C Becker, D Goldman, M Heilig, V A Ramchandani, L Leggio

**Affiliations:** 1 Section on Clinical Psychoneuroendocrinology and Neuropsychopharmacology, Laboratory of Clinical and Translational Studies, National Institute on Alcohol Abuse and Alcoholism, National Institutes of Health, Bethesda, MD, USA; 2 Department of Pharmacology, The Sahlgrenska Academy at University of Gothenburg, Gothenburg, Sweden; 3 Section on Human Psychopharmacology, Laboratory of Clinical and Translational Studies, National Institute on Alcohol Abuse and Alcoholism, National Institutes of Health, Bethesda, MD, USA; 4 Department of Psychiatry, Virginia Institute for Psychiatric and Behavioral Genetics, Virginia Commonwealth University, Richmond, VA, USA; 5 Laboratory of Clinical and Translational Studies, National Institute on Alcohol Abuse and Alcoholism, National Institutes of Health, Bethesda, MD, USA; 6 Intramural Research Program, National Institute on Drug Abuse, National Institutes of Health, Baltimore, MD, USA; 7 Section on Brain Electrophysiology and Imaging, Laboratory of Clinical and Translational Studies, National Institute on Alcohol Abuse and Alcoholism, National Institutes of Health, Bethesda, MD, USA; 8 Laboratory of Neurogenetics, National Institute on Alcohol Abuse and Alcoholism, National Institutes of Health, Bethesda, MD, USA; 9 Division of Neuroscience and Behavior, National Institute on Alcohol Abuse and Alcoholism, Bethesda, MD, USA; 10 Charleston Alcohol Research Center, Department of Psychiatry and Behavioral Science, Medical University of South Carolina, Charleston, SC, USA; 11 Department of Neurosciences, Medical University of South Carolina, Charleston, SC, USA; 12 Ralph H Johnson VA Medical Center, Charleston, SC, USA; 13 Center for Alcohol and Addiction Studies, Department of Behavioral and Social Sciences, Brown University, Providence, RI, USA

## Abstract

The hormone glucagon-like peptide-1 (GLP-1) regulates appetite and food intake. GLP-1 receptor (GLP-1R) activation also attenuates the reinforcing properties of alcohol in rodents. The present translational study is based on four human genetic association studies and one preclinical study providing data that support the hypothesis that GLP-1R may have a role in the pathophysiology of alcohol use disorder (AUD). Case–control analysis (*N*=908) was performed on a sample of individuals enrolled in the National Institute on Alcohol Abuse and Alcoholism (NIAAA) intramural research program. The Study of Addiction: Genetics and Environment (SAGE) sample (*N*=3803) was used for confirmation purposes. *Post hoc* analyses were carried out on data from a human laboratory study of intravenous alcohol self-administration (IV-ASA; *N*=81) in social drinkers and from a functional magnetic resonance imaging study in alcohol-dependent individuals (*N*=22) subjected to a Monetary Incentive Delay task. In the preclinical study, a GLP-1R agonist was evaluated in a mouse model of alcohol dependence to demonstrate the role of GLP-1R for alcohol consumption. The previously reported functional allele 168Ser (rs6923761) was nominally associated with AUD (*P*=0.004) in the NIAAA sample, which was partially replicated in males of the SAGE sample (*P*=0.033). The 168Ser/Ser genotype was further associated with increased alcohol administration and breath alcohol measures in the IV-ASA experiment and with higher BOLD response in the right globus pallidus when receiving notification of outcome for high monetary reward. Finally, GLP-1R agonism significantly reduced alcohol consumption in a mouse model of alcohol dependence. These convergent findings suggest that the GLP-1R may be an attractive target for personalized pharmacotherapy treatment of AUD.

## Introduction

Glucagon-like peptide-1 (GLP-1), signaling via its receptor (GLP-1R), has an important role in the gut–liver–brain axis. In addition to its role as a gut-derived incretin, GLP-1 acts as a neuropeptide, as it is produced by preproglucagon neurons in the nucleus of the solitary tract.^1^ Clinical studies show that GLP-1R is involved in food-related reward processing by acting as a meal termination signal.^2^ GLP-1R mRNA and protein are widely expressed in the human and rat brain.^3, 4^ In fact, the receptor gene is expressed in mesolimbic areas involved with reward processing in both species, including the globus pallidus, ventral tegmental area and nucleus accumbens.^3, 4^ The latter areas are innervated directly by GLP-1-producing neurons projecting from the nucleus of the solitary tract^5^ providing a route by which GLP-1 may affect motivation-related mechanisms.

The GLP-1R agonist, exendin-4, was recently shown to attenuate the reinforcing properties of alcohol in rodents,^6, 7^ by preventing alcohol-induced accumbal dopamine release.^6^ Conversely, the GLP-1R antagonist, exendin-9-39, increased alcohol intake in rats.^7^ These studies suggest a role of GLP-1R in alcohol use disorder (AUD), which represents the main hypothesis underlying this study.

Although preclinical research to date has suggested a potential role of GLP-1R in AUD, there is no human evidence to support this. Given that AUD is partially heritable, with contributions from numerous, yet to be established, loci,^8^ studying genetic variation of a pathway of interest, like GLP-1R in our case, is a possible approach. Specifically, we present five complementary studies, that is, four human genetic association studies investigating the genetics of GLP-1R in the context of AUD, and one preclinical study examining the role of GLP-1R agonism in a mouse model of alcohol dependence. First, we tested whether genetic variation in *GLP1R* is associated with AUD in participants enrolled at the National Institute on Alcohol Abuse and Alcoholism Laboratory of Clinical and Translational Studies (LCTS). Significant associations were further investigated for confirmatory purposes in the Study of Addiction: Genetics and Environment (SAGE) genome-wide association study sample. *Post hoc* analyses were conducted from a human laboratory study to explore the effects of identified *GLP1R* risk alleles on intravenous alcohol self-administration (IV-ASA), to provide initial functional validation of our hypothesis. As genetic variations are associated with changes in brain activity,^9^ further *post hoc* analyses were made using functional magnetic resonance imaging (fMRI) data to explore the effect of *GLP1R* risk alleles in alcohol-dependent individuals. Finally, the preclinical study investigated GLP-1R agonism on alcohol consumption as a pharmacological validation of the role of GLP-1R in alcohol dependence.

## Materials and methods

### Study procedure

#### LCTS study (Study 1)

Participants in the LCTS study were a subset (*N*=908) of a larger sample (*N*=1068) recruited at the National Institutes of Health (NIH) Clinical Center in Bethesda, MD, USA under two NIH Institutional Review Board-approved screening and evaluation protocols. Participants provided written informed consent. Cases comprised individuals that had either past and/or current AUD (*N*=669), while controls had no past or current alcohol and/or substance use disorder (*N*=239). AUD was diagnosed based on criteria for alcohol abuse and dependence using the SCID (Structured Clinical Interview for DSM-IV-TR Axis I Disorders).^10^ Lifetime psychotic disorders, but not other psychiatric comorbidity, were exclusionary. To avoid potential confounds from population stratification, the analysis was limited to the two major self-reported ancestries included in the sample, that is, Caucasian and African American (Table 1), thus excluding subjects of mixed, Asian or unknown descent (*N*=104). Control subjects that had a past or current substance use disorder (other than nicotine) were also excluded (*N*=11), as were subjects with missing SCID data (*N*=50).

#### SAGE study (Study 2)

For confirmation, we used the SAGE genome-wide association study, whose sample of alcohol-dependent and nondependent control individuals has been described in detail previously.^11, 12^ SAGE is a dbGaP study (accessions phs000092.v1.p1.c1 and c2), part of the Gene Environment Association Studies consortium. The sample was selected from the Collaborative Study on the Genetics of Alcoholism, the Family Study of Cocaine Dependence and the Collaborative Genetic Study of Nicotine Dependence data sets.

A lifetime history of alcohol dependence was diagnosed using DSM-IV criteria. Some control subjects met criteria for nicotine dependence on the basis of the Fagerström Test for Nicotine Dependence, but none had alcohol or any other substance dependence. Controls who endorsed ⩾3 DSM-IV symptoms of alcohol dependence, but did not cluster within a 12-month period, were removed as they may still have an increased genetic risk (*N*=55).^12^ *GLP1R* single-nucleotide polymorphisms (SNPs) were extracted using PLINK (version 1.07).^13^ Duplicate cases were removed, as were related subjects before analysis was initiated. The sample characteristics of the combined sample of alcohol-dependent cases (*N*=1917) and controls (*N*=1886) are seen in Table 2. The Institutional Review Boards at all participating sites approved data collection.

#### IV-ASA study (Study 3)

Male and female 21–44-year-old nondependent drinkers (*N*=81) were recruited for an NIH Institutional Review Board-approved human laboratory study with the IV-ASA paradigm (Table 3). Participants provided written informed consent.

The IV-ASA method, in which participants self-administer IV alcohol upon pressing a button, has the advantage over oral alcohol exposure to provide precision in terms of breath alcohol concentration (BrAC) exposure, and by assessing self-administration behavior driven primarily by the pharmacological effects of alcohol.^14^ The IV-ASA experiment consisted of two phases (details in Stangl *et al.*^15^). During the priming phase (25 min), participants were prompted to push the button to receive four small standardized alcohol infusions resulting in a BrAC level of ~30 mg% at 10 min for all participants. During the *ad libitum* phase (‘open-bar'), the participants were free to press the button any time they wished to receive the standardized IV alcohol infusion. The infusion rates were determined using the subject's age, height, weight and gender in a physiologically based pharmacokinetic model,^16^ and implemented using the Computerized Alcohol Infusion System.^17^ Each button press resulted in a 7.5 mg% increase in BrAC at a fixed rate of 3 mg% per minute for a fixed duration of 2.5 min followed by a 1 mg% decrease per minute until the next button press. The button was inactivated (with the participant's knowledge) if the next push passed the pre-set upper limit for BrAC exposure (100 mg%). An Alcotest 7410 handheld breathalyzer (Draeger Safety Diagnostics, Irving, TX, USA) was administered approximately every 15 min during the session.

#### fMRI study (Study 4)

Alcohol-dependent patients (*N*=22) from an Institutional Review Board-approved study^18^ were included in this analysis. Participants provided written informed consent. Participants were inpatients undergoing treatment for alcohol dependence at the NIH Clinical Center. They were scanned at least 1 week, but not more than 4 weeks after alcohol abstinence;^18^ fMRI was performed using a modified version of the Monetary Incentive Delay task.^18^

All the images were acquired in a 3 T MRI scanner (General Electric, Milwaukee, WI, USA); for the anatomical, a T1-weighted sequence was used (TE=2.5 ms, TR=6 ms, 0.94 × 0.94 × 1.2 mm^3^), and for the fMRI, a EPI-GRE single-shot sequence was used with the following parameters: TE=19.9 ms, TR=1 s, 16 contiguous sagittal slices centering on the intrahemispheric fissure, flip angle=90 °, average 3.75 × 3.75 mm^2^ in plane resolution, slice thickness=4 mm, 1 mm gap, for a total of 500 (volumes) seconds.

A total of 54 trials were randomly presented across three scanning runs. Each trial was composed of four events: (1) anticipatory cue (three levels: $0 (represented by a triangle), $1 (circle with one middle line) and $10 (circle with three lines)); (2) target (white square) for active response; (3) notification displaying the word ‘hit' (successful trial is indicated) or the world ‘win' (successful trial is indicated and rewarded); and (4) feedback on the trial outcome (amount earned in the trial and total earned until the present moment in the study). Participants were instructed to respond on a button box during the presentation of the target event. Stimuli were projected on a screen at the foot of the scanner bed and viewed using a head coil mirror. For additional details, see Bjork *et al.*^18^

#### Genotyping analyses (Studies 1–4)

Genotyping for Studies 1, 3 and 4 was performed at the National Institute on Alcohol Abuse and Alcoholism Laboratory of Neurogenetics. Genomic DNA was extracted from whole blood using standard protocols. DNA samples were genotyped using the Illumina OmniExpress BeadChip array (Illumina, San Diego, CA, USA) including more than 700 000 SNPs. The average genotype reproducibility was 0.99994. *GLP1R* SNPs, locations spanning from the 5′ to the 3′ flanking regions, were selected from the Illumina data set for analysis. The LCTS sample was split according to self-reported ancestry and deviation from Hardy–Weinberg Equilibrium was assessed only in control subjects for all 40 SNPs located in *GLP1R*. SNPs with a Hardy–Weinberg Equilibrium *P*-value <0.01 and a minor allele frequency of ⩽5% in Caucasian and/or African Americans were excluded from the analysis. We also excluded rs10305518 as this SNP was in perfect linkage disequilibrium (*r*^2^=1) with rs10305514 leaving a total of 26 SNPs (Supplementary Table S1). For Study 2, samples were genotyped on the Illumina Human 1 M Beadchip by the Center for Inherited Disease Research at Johns Hopkins University.^11^

#### GLP-1R agonist in a mouse model of alcohol dependence (Study 5)

The effects of AC3174, a GLP-1R agonist, on voluntary alcohol consumption were evaluated in a mouse model of alcohol dependence. Adult male C57BL/6 mice were obtained from Jackson Laboratories (Bar Harbor, ME, USA), individually housed and maintained in an AAALAC-approved facility under a modified 12-h light–dark cycle (lights on at 0200 h). Food and water were continuously available throughout the study. The study was conducted at the Medical University of South Carolina, approved by the Medical University of South Carolina Institutional Animal Care and Use Committee and consistent with the NIH Guide for the Care and Use of Laboratory Animals.

Study drug: AC3174 ([Leu14]exendin-4) is an exenatide analog with a single amino-acid substitution, leucine for methionine at position 14, done to eliminate the potential oxidation at methionine and potentially improve shelf-life stability.^19^ AC3174 binds to the GLP-1R *in vitro* and shares many of the biological and glucoregulatory activities of exenatide and GLP-1 *in vivo*. For additional details on the pharmacological profile of the compound, see Hargrove *et al.*^19^ For this study, four doses of AC3174 were examined, that is, 0, 0.03, 0.10 and 0.30 μg kg^−1^.

Study design: The general study design for the model of alcohol dependence and relapse drinking was similar to that previously reported.^20, 21^ Briefly, mice were first trained to drink alcohol (15% v/v) in a two-bottle choice (water as the alternative fluid) limited access (2 h per day) procedure. Voluntary drinking sessions started 30 min before the start of the dark phase of the circadian cycle. Once stable baseline drinking was established, mice were separated into two groups. One group (EtOH group) received repeated weekly cycles of chronic intermittent exposure (16 h per day for 4 days) to alcohol vapor in inhalation chambers. The remaining mice (CTL group) were similarly treated, but maintained in control (air) inhalation chambers. After a 72 h forced-abstinence period following each weekly inhalation exposure cycle, EtOH and CTL mice were given the opportunity to voluntarily drink alcohol under the same limited access conditions as baseline for five consecutive days. Thus, as depicted in Supplementary Figure S2, each weekly chronic intermittent alcohol (or air) exposure cycle was followed by a 5-day limited access drinking test cycle, and this pattern of treatment was repeated for several cycles. During baseline and test cycles 1–4 drinking sessions, all the mice received intraperitoneal injections of vehicle (saline) 15 min before access to alcohol to habituate them to this manipulation. During test cycles 5, 6 and 7, EtOH and CTL mice were further divided (*N*=9–10 per group) to receive intraperitoneal injections of saline or AC3174 (0.03, 0.10, 0.30 μg kg^−1^) 15 min before limited access drinking test sessions. Finally, mice received an additional two exposure cycles and during test cycles 8 and 9, all the mice were injected with vehicle before each drinking session. These testing periods served as a drug washout evaluation to test for any long-lasting effect of treatment on voluntary alcohol intake (see Supplementary Figure S2).

### Statistical analyses

#### LCTS study (Study 1)

For the case–control study, association between genotype and affection status were calculated using logistic regression controlling for ancestry (self-reported) and determined by the odds ratio and the corresponding 95% confidence interval. An additive model was assumed, coding the genotype as 0, 1 or 2 depending on the number of minor alleles that were present. The odds ratio thus represents the odds for AUD associated with each copy of the minor allele. To adjust for multiple testing, the MeffLi (effective number of independent marker loci) method, a correction method taking into account the correlation between the studied SNPs, was applied using the online software SNPSpD (available at http://gump.qimr.edu.au/general/daleN/SNPSpD).^22, 23^ Separate logistic regression analyses were also carried out within self-reported ancestral categories. Haplotype-based analyses were also conducted for Study 1 and 2 (see Supplementary Information).

Nicotine dependence and AUD are highly comorbid,^24^ and smoking-related variables correlate with alcohol dependence severity.^25^ We, therefore, carried out *post hoc* analyses to evaluate the association of the GLP1R markers with smoking status. For this analysis, participants were divided into three groups: non-smoking controls, non-smoking subjects with AUD and smoking subjects with AUD. Few control subjects were smokers and this group was thus excluded from the analysis. Comparisons between allele frequencies in the three groups were investigated in self-reported Caucasians and African Americans separately using *χ*^2^ test and Cochran–Armitage trend test.

#### SAGE study (Study 2)

SNPs nominally associated with AUD in the LCTS study were tested for association with alcohol dependence in the whole SAGE sample as well as in males and females separately by logistic regression controlling for ancestry using principal component factors, PC1 and PC2, with PC1 distinguishing between European and African ancestry and PC2 distinguishing between Hispanic and non-Hispanic subjects.

#### IV-ASA study (Study 3)

We carried out *post hoc* analyses of the human laboratory data to examine whether risk alleles identified in the case–control study were also associated with measures of alcohol self-administration in the IV-ASA experiment: peak BrAC, average BrAC, total number of rewards, total alcohol self-administered in grams and number of subjects reaching a BrAC of 80 mg% (corresponding to a binge exposure). Outcomes were analyzed using linear or logistic regression models, controlling for age, body mass index, gender and ancestry, with genotype evaluated using an additive model (coded as 0, 1 or 2 depending on the number of minor alleles).

Statistical analyses for studies 1–3 were carried out using IBM SPSS statistics for Windows (version 20.0.0, IBM, Armonk, NY, USA) and PLINK.^13^

#### fMRI study (Study 4)

The fMRI analyses were carried using the Analysis of Functional NeuroImages software.^26^ The three fMRI runs were concatenated, time shifted, motion corrected, warped into Talairach space as 3.5 mm isotropic voxels and smoothed to a full width at half maximum of 6 mm. Processed time series for each subject were then modeled with canonical hemodynamic responses time-locked to anticipatory cues of high reward, low reward and neutral conditions with the respective trial outcome notifications (feedback); in addition, head motion parameters were also entered into the model as regressor of no interest. The following linear contrasts were generated and used for group analysis: cue of high ($10)/low ($1) reward, cue of high/low reward vs neutral ($0), the respective feedbacks alone and vs the neutral feedback condition. Canonical hemodynamic responses and time-series data sets were scaled so that beta weights could be interpreted as percent-signal-change. A voxel-wise, between-group comparison for the entire brain was carried out using Multivariate Modeling (3dMVM),^27^ with one contrast per condition and per subject, including subject's self-reported ancestry as a categorical factor. Results were corrected for multiple comparison at a small volume of interest using the 3dClustSim program in Analysis of Functional NeuroImages (v.2011) by generating 1000 Monte Carlo simulations on a volume 8918 mm^3^, centered in the activated cluster, using 8 mm full width at half maximum of smoothing and voxel size of 3.5 × 3.5 × 3.5 mm^3^ to determine the cluster size at which the false positive probability was below a desired alpha level of *P*_corr_<0.05. Average percent BOLD signal change (region of interest (ROI) analysis) was obtained from the beta weights generated for each task condition for a small spherical volume (8 mm diameter) centered at the peak-activated voxel of the significant clusters (right globus pallidus) as well as the left globus pallidus, left and right nucleus accumbens and ventral tegmental area and then analyzed using *t*-test.

#### GLP-1R agonism in a mouse model of alcohol dependence (Study 5)

Preliminary analyses of the data indicated that there were no significant variations in alcohol intake across days during each test cycle. Therefore, alcohol intake data (g kg^−1^) were averaged over 5 days of testing and analyzed by factorial analysis of variance (ANOVA) with group (EtOH vs CTL) and treatment (0, 0.03, 0.10, 0.30 μg kg^−1^ AC3174) as main factors for each testing period.

## Results

### LCTS study (Study 1)

#### Single marker associations

The 26 SNPs for the whole case–control sample (that is, both Caucasian and African American subjects) were submitted to SNPSpD and gave a MeffLi of 16.0 and a significance threshold of *P*<0.0032. Significant associations with affection status were observed for four SNPs (Table 4). *Post hoc* analysis was performed to investigate whether these results were replicable across ancestral groups. Despite large differences in allele frequencies (Supplementary Table S2) and a greatly underpowered African American sample, replications (rs7766663, rs2235868 and rs7769547) or a trend-level replication (rs10305512) were found for all four SNPs in both ancestral groups (Table 4). See Supplementary Information for further results on single maker and haplotype analyses.

#### *Post hoc* analyses

**Figure 1:**
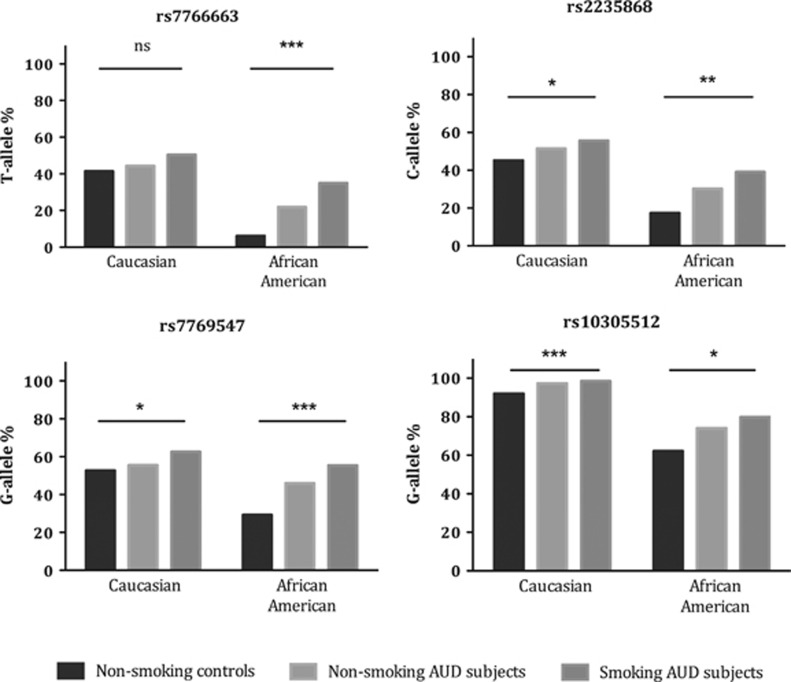
Comparison of risk allele frequencies between non-smoking controls, non-smoking subjects with alcohol use disorder (AUD) and smoking subjects with AUD in the LCTS study using Pearson *χ*^2^ test. Total *n* for the three groups were 125–130, 121–128, 202–215 for the Caucasian sample and 26–29, 47–50, 137–147 for the African American sample, respectively. *P<0.05, **P<0.01, ***P<0.005. LCTS, Laboratory of Clinical and Translational Studies.

We conducted *post hoc* analyses with the four associated SNPs and the two functional SNPs included in the haplotype block (see Supplementary Information). Comparing allele frequencies among non-smoking controls, non-smoking AUD and smoking AUD subjects using a *χ*^2^ test revealed that the non-smoking AUD group formed an intermediate group with the highest risk allele frequencies in the smoking AUD group (Figure 1). This was replicated across the two ancestral groups and seen for three of the six SNPs (Caucasians: rs2235868 *P*=0.030, rs7769547 *P*=0.026, rs10305512 *P*<0.001; African-Americans: rs2235868 *P*=0.0043, rs7769547 *P*<0.001, rs10305512 *P*=0.012). In addition, the results of the *χ*^2^ analyses suggested linear trends in allele frequencies across the three groups. Consequently, we applied the Cochran–Armitage test and found that the results remained significant for all SNPs, with *P*-values even smaller than those reported for the *χ*^2^ analyses. To investigate whether non-smoking AUD and smoking AUD subjects differed in AUD severity, we performed a linear regression using group as the independent factor, and, as the dependent factor, we used number of heavy drinking days (defined as ⩾4/5 drinks per day for women/men) during a 90-day-period as reported in the timeline follow-back self-report questionnaire,^28^ controlling for age and gender. We found significantly more heavy drinking days in the smoking AUD subjects (F(3,568)=13.5, *B*=11.6, *P*<0.001).

### SAGE study (Study 2)

None of the SNPs nominally associated with AUD in the LCTS study were associated with affection status in SAGE when the whole sample was investigated. However, when the sample was split by gender, a logistic regression controlling for PC1 and PC2 showed that the 168Ser/Ser genotype was associated with alcohol dependence in males (*β*=0.190, s.e.=0.089, *N*=1752, *P*=0.033) but not in females (*β*=−0.072, s.e.=0.078, *N*=2036, *P*=0.357). On the basis of these results, we reanalyzed the LCTS case–control results for the relevant SNPs splitting the sample by gender and found the same pattern with a more pronounced association in males (Supplementary Table S3). However, we also observed that association between rs7769547 and AUD in the LCTS sample was replicated in both genders, and there was a trend toward replication for three of the SNPs (rs7766663, rs2235868 and rs10305512). See Supplementary Information for further results involving haplotype analyses.

### IV-ASA study (Study 3)

Data from the IV-ASA study were analyzed against the SNPs nominally associated with AUD in Study 1. Significant associations were seen for rs6923761 with average BrAC, peak BrAC and the percent of subjects that reached a BrAC of 80 mg% (Table 5). Each of these measures was shown to increase with the addition of each 168Ser allele.

### fMRI study (Study 4)

On the basis of the results from Studies 1–3, the sample for Study 4 was divided according to rs6923761 genotype in which 168Ser (A) allele carriers were merged into one group: group 168Ser/Gly+168Ser/Ser (five males, five females; age: 31.5±6.3; ancestry: seven Caucasians, two African Americans and one unknown); and group 168Gly/Gly(six males, six females; age: 32.7±6.5; ancestry: seven Caucasians, four African American and one multiethnic ancestry). The groups did not differ for either gender or age.

**Figure 2:**
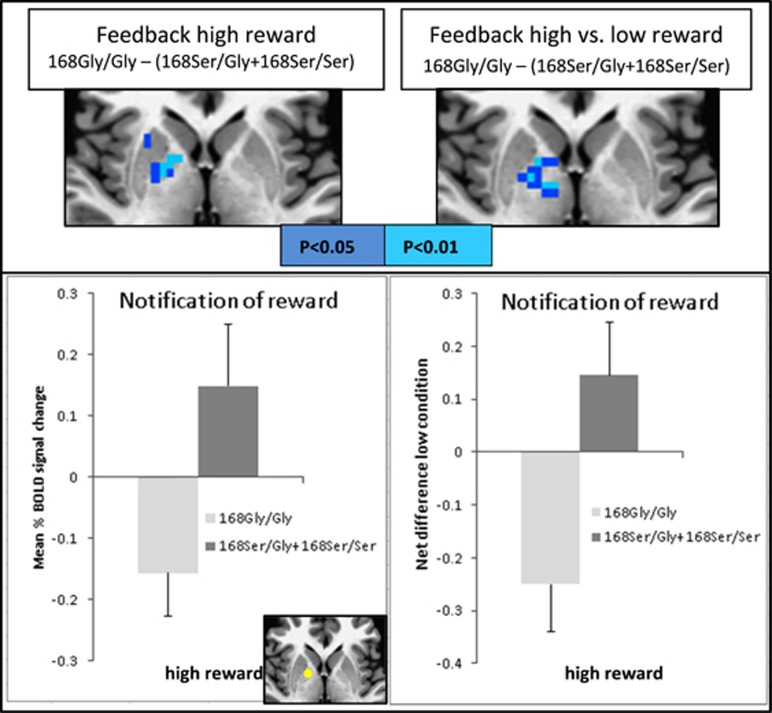
Statistical maps for fMRI analysis contrasting the genotype groups 168Gly/Gly × 168Ser/Gly+168Ser/Ser in the rs6923761 SNP. Statistical maps (top) and ROI results (bottom) for notification of high reward (left) and its net difference with low reward (right) contrasting (168Gly/Gly)−(168Ser/Gly+168Ser/Ser) are shown; ROI localization is displayed in yellow at the axial view in the bottom. fMRI, functional magnetic resonance imaging; SNP, single-nucleotide polymorphism.

There was a significant difference (*P*_corr_<0.05) in brain activation within the right globus pallidus when contrasting the genotypes (168Gly/Gly) vs (168Ser/Gly+168Ser/Ser) for the rs6923761 SNP. Those carrying the non-risk allele (that is, 168Gly/Gly group) had lower BOLD response than those carrying the risk allele (168Ser/Gly+168Ser/Ser group) when receiving notification of outcome (feedback) for high reward (peak *T*=−4.8). A similar group difference was observed for the contrasted task condition: notification of outcome of high reward vs low reward (peak *T*=−3.4). We then computed the mean percent signal change of ROIs as described above to further verify these findings. The only significant difference was found from the ROI located on right globus pallidus for high reward between group contrast (Figure 2). No significant group difference was observed for other task conditions (contrasts) or other brain areas under the same contrast.

### GLP-1R agonism in a mouse model of alcohol dependence (Study 5)

**Figure 3:**
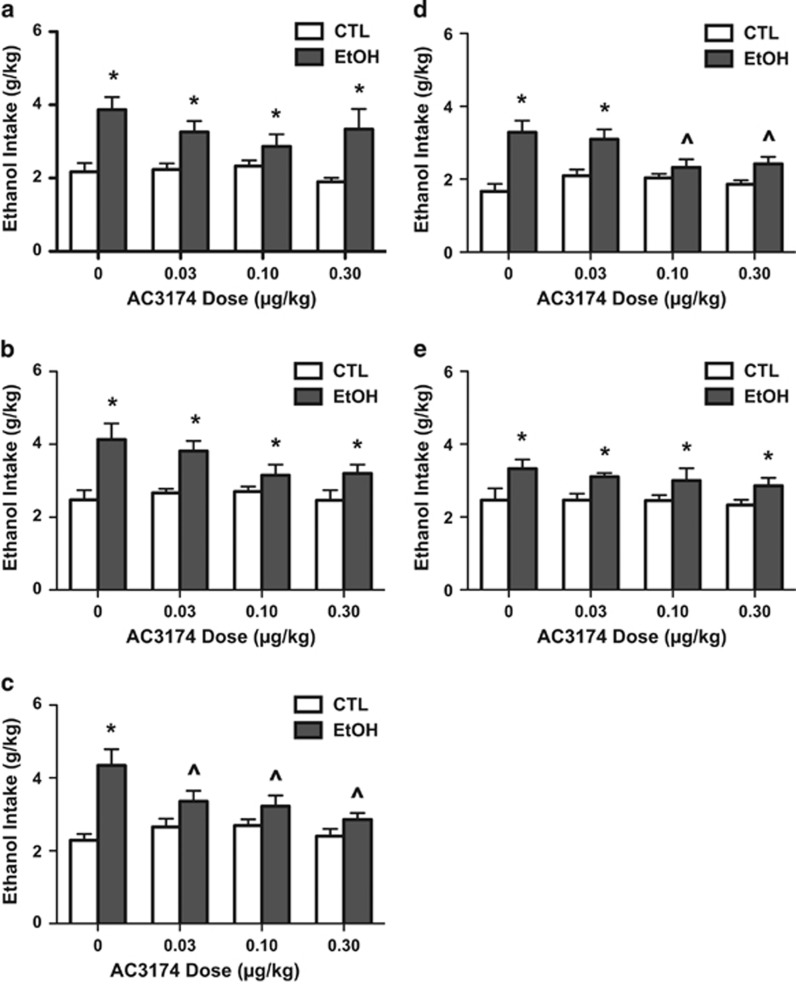
GLP-1R agonism in a mouse model of alcohol dependence. (**a**) AC3174 treatment #1 (alcohol intake during test cycle 5): EtOH mice consumed significantly more alcohol than CTL mice (*P*< 0.001). Analysis of variance (ANOVA) failed to indicate a main effect of treatment or group × treatment interaction. (**b**) AC3174 treatment #2 (alcohol intake during test cycle 6): there was a significant main effect of group (*P*<0.001), with EtOH mice consuming a greater amount of alcohol than CTL mice. ANOVA failed to indicate a main effect of treatment or group × treatment interaction. (**c**) AC3174 treatment #3 (alcohol intake during test cycle 7): ANOVA indicated a significant main effect of group (*P*<0.00001) and a significant group × treatment interaction (*P*<0.025). *Post hoc* comparisons indicated that, as expected, EtOH mice injected with vehicle consumed more alcohol than CTL mice. In addition, all doses of AC3174 significantly reduced drinking compared with the vehicle condition in EtOH mice, while AC3174 treatment did not significantly alter alcohol intake in nondependent CTL mice. Further, AC3174 treatment abolished the difference in alcohol intake between EtOH and CTL conditions. (**d**) Placebo (washout) test #1 (alcohol intake during test cycle 8): all mice were treated with saline (drug-washout test) to substantiate the apparent efficacy of AC3174 to reduce escalated alcohol drinking in dependent mice. ANOVA revealed a significant main effect of group (*P*<0.00001) and a significant group × treatment interaction (*P*<0.01). *Post hoc* comparisons supported the expected greater alcohol intake in EtOH compared with CTL mice that continued to receive vehicle. A similar profile of results was obtained in mice that received the lowest AC3174 dose (0.03 μg kg^−1^) in the previous test cycle. However, EtOH mice that received 0.10 or 0.30 μg kg^−1^ AC3174 doses in the previous test period continued to consume significantly less alcohol compared with mice that previously received vehicle, and their lower level of intake was similar to that exhibited by the corresponding CTL groups. (**e**) Placebo (washout) test #2 (alcohol intake during test cycle 9): ANOVA indicated a significant main effect of group (*P*<0.001), but no effect of treatment or an interaction between group × treatment during this second washout test period. These results indicate that after a second week of placebo (saline) treatment, elevated drinking in EtOH compared with CTL mice was restored in all the test groups. *P<0.05, significantly different from corresponding CTL group; ^*P*<0.05, significantly different from corresponding vehicle group.

Consistent with previously published work,^20, 21^ EtOH mice showed significant progressive escalation of voluntary alcohol intake over their baseline intake, compared with CTL mice, whose intake remained relatively stable over successive four exposure/test cycles. Intake data during the last test cycle before AC3174 treatment indicated a significant difference between EtOH and CTL mice (F(1,67)=13.61, *P*<0.0001). Analysis indicated that EtOH mice consumed significantly more alcohol than CTL mice, and this effect did not differ at baseline (that is, before drug administration) across the different treatment groups (F(3,67)<1.0; *P*, not significant). Results indicate that AC3174 significantly reduced alcohol consumption in a mouse model of alcohol dependence (Figures 3a–e).

#### AC3174 treatment #1 (alcohol intake during test cycle 5)

EtOH mice consumed significantly more alcohol than CTL mice (main effect of group (F(1,67)=31.80, *P*<0.001)). However, ANOVA failed to indicate a main effect of treatment or group × treatment interaction (Figure 3a).

#### AC3174 treatment #2 (alcohol intake during test cycle 6)

There was a significant main effect of group (F(1,67)=27.88, *P*<0.001), with EtOH mice consuming a greater amount of alcohol than CTL mice. Again, ANOVA failed to indicate a main effect of treatment or group × treatment interaction (Figure 3b).

#### AC3174 treatment #3 (alcohol intake during test cycle 7)

ANOVA indicated a significant main effect of group (F(1,65)=31.44, *P*<0.00001) and a significant group × treatment interaction (F(3,65)=4.01, *P*<0.025). *Post hoc* comparisons (Newman–Keuls test) indicated that, as expected, EtOH mice injected with vehicle consumed more alcohol than CTL mice. In addition, all doses of AC3174 significantly reduced drinking compared with the vehicle condition in EtOH mice, while AC3174 treatment did not significantly alter alcohol intake in nondependent CTL mice. Further, AC3174 treatment abolished the difference in alcohol intake between EtOH and CTL conditions (Figure 3c).

#### Placebo (washout) test #1 (alcohol intake during test cycle 8)

All mice were treated with saline (drug-washout test) to substantiate the apparent efficacy of AC3174 to reduce escalated alcohol drinking in dependent mice. ANOVA revealed a significant main effect of Group (F(1,64)=38.61, *P*<0.00001) and a significant group × treatment interaction (F(3,64)=4.14, *P*<0.01). *Post hoc* comparisons supported the expected greater alcohol intake in EtOH compared with CTL mice that continued to receive vehicle. A similar profile of results was obtained in mice that received the lowest AC3174 dose (0.03 μg kg^−1^) in the previous test cycle. However, EtOH mice that received 0.10 or 0.30 μg kg^−1^ AC3174 doses in the previous test period continued to consume significantly less alcohol compared with mice that previously received vehicle, and their lower level of intake was similar to that exhibited by the corresponding CTL groups (Figure 3d).

#### Placebo (washout) test #2 (alcohol intake during test cycle 9)

ANOVA indicated a significant main effect of group (F(1,64)=16.30, *P*<0.001), but no effect of treatment or an interaction between group × treatment during this second washout test period. These results indicate that after a second week of placebo (saline) treatment, elevated drinking in EtOH compared with CTL mice was restored in all the test groups (Figure 3e).

## Discussion

We report a set of studies which, taken together, support the hypothesis that GLP-1R has a role in AUD and represents a novel therapeutic target. First, we report evidence and replication that genetic variation in *GLP1R* is associated with AUD and alcohol dependence in humans. In addition to providing an internal replication by splitting the LCTS sample by ancestry, we confirmed the nominal association between AUD and rs6923761 in this sample by showing a similar association with alcohol dependence in males from an independent cohort (SAGE). Albeit exploratory in its nature, preliminary human functional validation was obtained from two studies, in which the *GLP1R* 168Ser allele was associated with increased measures of alcohol self-administration from the IV-ASA study, and significantly higher BOLD signal in an fMRI study at the globus pallidus when participants received rewarding feedback during the Monetary Incentive Delay task. Finally, we report that pharmacological GLP-1R agonism with AC3174 significantly reduced alcohol consumption in a mouse model of alcohol dependence.

GLP-1 influences appetite and reward function through peripheral and central actions on the GLP-1R.^29^ It decreases food intake in both humans and animals,^30^ a mechanism most likely to involve GLP-1R activation in the area postrema, central nucleus of the amygdala and nucleus of the solitary tract.^31, 32^ GLP-1Rs are also present in areas involved with reward processing such as globus pallidus, ventral tegmental area and nucleus accumbens. The nucleus of the solitary tract contains GLP-1-producing neurons,^1^ which project directly to the ventral tegmental area and nucleus accumbens.^5^ The nucleus of the solitary tract also receives vagal afferents from the stomach providing an additional mechanism through which peripherally secreted GLP-1 signals the brain.^1^

An *in vitro* study reported the effect on receptor function by one of the SNPs nominally associated with AUD in this study. Using cells expressing wild-type and polymorphic human *GLP1R*, the 168Ser (A-allele) was associated with decreased GLP-1R cell surface expression compared with their complementary alleles.^33^ Although the affinity for a range of GLP-1 agonists was unaffected by genotype, the efficacy of agonist response was lower for the 168Ser variant, presumably because of its lower expression.^33^ Seemingly in agreement with the *in vitro* findings, the 168Ser allele was associated with lower insulin response following GLP-1 infusion in healthy volunteers^34^ and with lower metabolic/cardiovascular biomarkers in obese females.^35^ Furthermore, a haplotype uniquely including the 168Ser allele was associated with significantly better response to perphenazine and worse response to olanzapine, providing support for potential pharmacogenetic interactions.^36^ In addition, the 260Phe allele (C) of Phe260Leu (rs1042044), which here was associated with AUD at a trend level, has previously been associated with significantly higher morning salivary cortisol levels in children compared with homozygotes for the 260Leu allele.^37^ This is consistent with the previously reported role of GLP-1 signaling in activating the hypothalamic–pituitary–adrenal axis, which in turn is important for the etiology of AUD.^38^

The gender differences reported here, with the rs6923761 being associated with alcohol dependence only in males of the SAGE cohort, find support in the literature. The prevalence of AUD is higher among men than women and twin pair correlations for AUD has been reported to be lower in opposite-sex dizygotic twins when compared with same-sex dizygotic twins.^39^ One of several possible explanations for the differences in genetic sources of liability is the fact that, while men and women share the same AUD susceptibility genes, there may still be sex differences in genetic background and/or gene–gene interaction.^39^ However, it is worth noting that a secondary analysis in the LCTS sample revealed further replications and trend-level replications across genders for some of the associated SNPs.

There is one main difference between the SAGE and the LCTS cohorts. In SAGE, all cases were alcohol dependent, and although cases in the LCTS cohort were mainly alcohol-dependent subjects, we also included alcohol abusers (*N*=59). Alcohol abuse and dependence, the two syndromes included in the AUD diagnosis, have a shared etiology,^40^ which is consistent with the fact that we were able to replicate our findings from LCTS in the male sample of SAGE.

The association between *GLP1R* risk alleles and AUD was most pronounced in individuals who were also smokers, a finding replicated across the two ancestral groups. Alcohol and nicotine addiction are frequently comorbid and have overlapping genetic vulnerability^41^ and neurobiological factors. Nicotine use among AUD subjects is also associated with a greater severity of alcohol dependence.^25^ This is consistent with our results, not only as the smoking AUD subjects had significantly more heavy drinking days than non-smoking AUD subjects, but we also found a graded increase in risk allele frequencies when comparing non-smoking control, non-smoking AUD and smoking AUD subjects. Notably, a preclinical study showed that GLP-1R agonism attenuates nicotine-induced effects.^42^ These findings hold potentially important clinical implications because alcoholic smokers have an increased risk of tobacco-related mortality and morbidity.^43^

Given the possible functional role of SNPs discussed above, coupled to the findings reported here, we hypothesize that variations in *GLP1R* may affect GLP-1 expression and signaling both peripherally and centrally. This, in turn, may moderate responses to alcohol, and/or motivation to consume it. As human genetic association studies are fraught with numerous potential confounds, we validated our findings using two human experimental paradigms conducted in rigorous and well-controlled conditions. As a first step toward functional validation in humans, we found that with increased number of the *GLP1R* 168Ser alleles, subjects self-administered alcohol to a higher level as indicated by increased average and peak BrAC and increased number of subjects reaching a BrAC of 80 mg%. This observation provides an intriguing, albeit preliminary, indication that the 168Ser *GLP1R* variant influences alcohol-drinking behavior, which in turn may predispose towards AUD as seen in the case–control analyses. We can only speculate on the mechanism underlying this relationship; however, given the previous *in vitro* functional report for the 168Ser allele,^33^ we postulate that decreased function of the SNP attenuates the regulatory properties of GLP-1R, including alcohol intake as demonstrated in preclinical studies,^6, 7^ (present Study 5) leading to alcohol self-administration and increased susceptibility for AUD. As a second step toward functional validation in humans, we found that alcohol-dependent individuals carrying the 168Ser allele had significantly higher BOLD signal than the homozygote group carrying the complementary allele at the globus pallidus when receiving notification of reward. Human neuroimaging studies have shown that the globus pallidus is related to reward processing,^44, 45^ which is consistent with preclinical studies indicating its involvement in reward via ventral striatal dopaminergic projections to the globus pallidus.^46^ Given the positive correlation between reward-related mesolimbic activations and striatal dopamine release,^47^ these fMRI results, though preliminary, suggest that those carrying the risk allele may have a more dysfunctional reward system that contributes to their higher vulnerability to AUD. Notably, the globus pallidus has a high concentration of GLP-1 binding sites in the human brain.^3^ Although speculative, these preliminary data are consistent with recent work suggesting that activation of GLP-1R modulates dopamine signaling,^6, 48, 49^ which in turn is dysregulated in AUD.^50^ Given the emerging importance of epigenetic modification in addictions,^51^ future research will also need to investigate the possible role of epigenetic mechanisms in regulating the GLP-1R gene in AUD.

The GLP-1R agonist, exendin-4, has previously been shown to reduce alcohol intake, alcohol-induced reward and the motivation to consume alcohol in rodents,^6, 7^ whereas the GLP-1R antagonist, exendin-9-39, increased alcohol intake in rats.^7^ Our *a priori* hypothesis was that the receptor rather than the peptide itself has a key role in alcohol-related seeking behaviors. Therefore, our human analyses were *a priori* limited to the gene encoding the receptor. Consistent with our hypothesis and the results obtained in these human experiments, we found converging evidence in a mouse model of alcohol dependence where pharmacological GLP-1R agonism via an exendin-4 analog (AC3174) significantly reduced alcohol consumption. Interestingly, in nondependent mice, AC3174 did not affect modest levels of voluntary alcohol consumption, nor was it effective in reducing elevated alcohol drinking in dependent mice during its first administration. However, with extended administration (over additional test cycles), AC3174 was effective in selectively reducing escalated drinking exhibited by dependent mice. This effect, once established, was relatively long lasting in mice receiving the higher doses. Only after a second washout test cycle did alcohol consumption in dependent mice return to pre-treatment alcohol intake levels, thus suggesting that the effects of AC3174 may manifest after chronic treatment.

Our reverse translation (Study 5) of the human work was conducted via a pharmacological probe rather than a knockout genetic model. This approach is more clinically relevant and therefore strengthens this set of studies. However, future studies are needed to fully understand the properties of AC3174 itself as a potential treatment for AUD, including locomotor activity, loss of righting reflex, pharmacokinetics/pharmacodynamics and operant procedure studies, as well as experiments addressing its specificity for alcohol intake (for example, compared with saccharine or sucrose intake, as well as control measures such as water and food intake). Though future studies will need to address these limitations, it is important to point out that the current preclinical study is in line with previous experiments in rodents,^6, 7^ which incidentally reported no effect of the GLP-1 analog exendin-4 on such control measures. The current study further adds novel important information, that is, (1) it provides evidence from a different animal model and lab; (2) it holds significant clinical relevance, given that the effects were specifically detected in a mouse model of alcohol dependence; and (3) it was not conducted with exendin-4 itself but rather with another analog, thus providing additional evidence toward our overall hypothesis on the specific role of the GLP-1R regardless of its ligand (for example, GLP-1, exendin-4 or AC3174) in excessive alcohol consumption.

There is a large need for effective pharmacotherapies for AUD that can be tailored to individual risk factors and/or also provide a biologically oriented treatment integrated with individualized psychotherapies.^38^ Notably, GLP-1 analogs are already approved and clinically used for the treatment of type 2 diabetes. Our study suggests the GLP-1R as a candidate treatment target, potentially with a particular utility in a genetically identified subpopulation of AUD patients.

## Figures and Tables

**Table 1:** Sample characteristics for the LCTS study

	*AUD*			*Controls*		
	*Total sample*	*Women*	*Men*	*Total sample*	*Women*	*Men*
*N* (%)	670 (100)	199 (29.7)	471 (70.3)	238 (100)	99 (41.6)	139 (58.4)
Age (s.d.)	41.2 (±10.7)	41.9 (±10.8)	40.9 (±10.6)	30.9 (±10.1)	31.4 (±9.8)	30.6 (±10.3)
						
*Self-reported ancestry*						
Caucasian	421 (62.8)	132 (66.3)	289 (61.4)	184 (77.3)	78 (78.8)	106 (76.3)
African American	249 (37.2)	67 (33.7)	182 (38.6)	54 (22.7)	21 (21.2)	33 (23.7)
						
*Self-reported ethnicity*						
Hispanics	9 (1.3)	4 (2.0)	5 (1.1)	6 (2.5)	3 (3.0)	3 (2.2)
Smokers	363 (54.2)	104 (52.3)	259 (55.0)	18 (7.6)	6 (6.1)	12 (8.6)
Current alcohol dependence	578 (86.3)	175 (87.9)	403 (85.6)	0	0	0
Past alcohol dependence	169 (25.2)	53 (26.6)	117 (24.8)	0	0	0
Current alcohol abuse	21 (3.1)	4 (2.0)	17 (3.6)	0	0	0
Past alcohol abuse	43 (6.4)	11 (5.5)	32 (6.8)	0	0	0
						
*Lifetime psychiatric comorbidity*^a^						
Mood disorder	131 (19.6)	60 (30.2)	71 (15.1)	10 (4.2)	4 (4.1)	6 (4.3)
Anxiety disorder	245 (36.6)	101 (50.8)	144 (30.6)	9 (3.8)	5 (5.2)	4 (2.9)
Substance use disorder^b^	435 (64.9)	114 (57.3)	321 (68.2)	0	0	0
Eating disorder	16 (2.4)	14 (7.0)	2 (0.4)	0	0	0

Abbreviations: AUD, alcohol use disorder; LCTS, Laboratory of Clinical and Translational Studies.

a Complete Structured Clinical Interview for DSM-IV-TR Axis I Disorders (SCID) available for 665 cases and 234 controls.

b Not including alcohol use disorder.

Data are presented as *N* (%) unless otherwise specified.

**Table 2:** Sample characteristics for the SAGE study

	*Alcohol dependent*			*Controls*^a^		
	*Total*	*Women*	*Men*	*Total*	*Women*	*Men*
*N* (%)	1917 (100)	746 (38.9)	1171 (61.1)	1886 (100)	1299 (68.9)	587 (31.1)
Age (s.d.)	39.0 (9.3)	37.9 (8.3)	39.7 (9.8)	39.3 (9.1)	39.4 (8.8)	39.2 (9.9)
						
*Self-reported ancestry*						
Caucasian	1244 (64.9)	483 (64.7)	761 (65.0)	1391 (73.5)	975 (74.8)	416 (70.6)
African American	671 (35.0)	261 (35.0)	410 (35.0)	495 (26.1)	324 (24.8)	171 (29.0)
						
*Self-reported ethnicity*						
Hispanics	78 (4.1)	21 (2.8)	57 (4.9)	60 (3.2)	38 (2.9)	22 (3.7)
Nicotine dependence	1311 (68.4)	548 (73.5)	763 (65.2)	369 (19.5)	292 (22.4)	77 (13.1)

Abbreviation: SAGE, Study of Addiction: Genetics and Environment.

a Not including controls who endorsed ⩾3 DSM-IV symptoms of alcohol dependence, but did not cluster within a 12-month period (*N*=55).

Data are presented as *N* (%) unless otherwise specified.

**Table 3:** Sample characteristics for the IV-ASA study

	*Total sample*	*Women*	*Men*
*N* (%)	84 (100)	47 (56.0)	37 (44.0)
Age (s.d.)	25.5 (4.2)	25.6 (4.2)	25.2 (4.3)
			
*Self-reported ancestry*			
Caucasian	72 (85.7)	39 (83.0)	33 (89.2)
African American	12 (14.3)	8 (17.0)	4 (10.8)
			
*Self-reported Ethnicity*			
Hispanics	4 (4.8)	2 (4.3)	2 (5.4)
Smokers^a^	5 (6.9)	4 (10.3)	1 (3.0)
Current alcohol dependence	0	0	0
Past alcohol dependence	0	0	0
Current alcohol abuse	0	0	0
Past alcohol abuse	5 (6.0)	5 (10.6)	0
			
*Lifetime psychiatric comorbidity*^b^			
Mood disorder	5 (2.9)	4 (8.7)	1 (2.7)
Anxiety disorder	4 (5.8)	2 (5.4)	2 (6.3)
Substance use disorder^c^	0	0	0
Eating disorder	0	0	0

Abbreviation: IV-ASA, intravenous alcohol self-administration.

a Smoking status reported by 72 subjects.

b Complete Structured Clinical Interview for DSM-IV-TR Axis I Disorders (SCID) available for 83 subjects.

c Not including alcohol use disorder.

Data are presented as *N* (%) unless otherwise specified.

**Table 4:** Genetic association testing between *GLP1R* SNPs and alcohol use disorder

*GLP1R SNP*	*Minor allele/major allele*	*Caucasian and African American*			*Caucasian*			*African American*		
		N	*OR (95% CI)*	P*-value*^a^	N	*OR (95% CI)*	P-*value*^b^	N	*OR (95% CI)*	P-*value*^b^
rs7738586	A/C	904	0.76 (0.54–1.05)	0.0983	602	0.76 (0.51–1.12)	0.1597	302	0.75 (0.39–1.44)	0.3836
rs9296274	G/A	904	0.81 (0.60–1.09)	0.1678	602	0.88 (0.60–1.30)	0.5211	302	0.71 (0.45–1.14)	0.1601
rs2268657	C/T	901	0.98 (0.78–1.22)	0.8429	600	1.00 (0.78–1.29)	0.9969	301	0.91 (0.58–1.43)	0.6871
rs3799707	T/G	902	1.07 (0.84–1.38)	0.5753	601	1.03 (0.79–1.35)	0.8081	301	1.34 (0.69–2.60)	0.3917
rs10305439	A/C	903	1.13 (0.90–1.43)	0.2910	603	1.10 (0.86–1.42)	0.4442	300	1.29 (0.72–2.32)	0.3881
rs2143734	G/A	903	0.79 (0.63–0.99)	0.0412	602	0.82 (0.63–1.07)	0.1539	301	0.68 (0.43–1.09)	0.1078
rs2268650	A/G	902	1.31 (1.02–1.68)	0.0345	600	1.28 (0.98–1.67)	0.0666	302	1.55 (0.72–3.33)	0.2664
rs910170	A/G	904	0.85 (0.69–1.06)	0.1443	603	0.89 (0.69–1.13)	0.3359	301	0.76 (0.50–1.17)	0.2116
rs874900	G/A	850	0.76 (0.53–1.07)	0.1161	561	0.53 (0.32–0.88)	0.0146	289	1.03 (0.63–1.67)	0.9148
rs6923761	A/G	904	1.46 (1.12–1.89)	0.0047	602	1.45 (1.10–1.90)	0.0083	302	1.54 (0.67–3.59)	0.3119
**rs7766663**	**T/G**	842	**1.42 (1.13**–**1.79)**	**0.0032**	565	**1.32 (1.02**–**1.72)**	**0.0365**	277	**1.90 (1.11**–**3.28)**	**0.0202**
rs7341356	G/A	847	1.43 (1.12–1.81)	0.0035	568	1.35 (1.03–1.77)	0.0296	279	1.72 (1.03–2.86)	0.0379
**rs2235868**	**C/A**	904	**1.49 (1.18**–**1.87)**	**0.0006**	602	**1.43 (1.10**–**1.86)**	**0.0070**	302	**1.70 (1.05**–**2.74)**	**0.0311**
rs1042044	A/C	904	0.81 (0.65–1.00)	0.0512	604	0.84 (0.65–1.07)	0.1617	300	0.73 (0.48–1.11)	0.1448
rs932443	C/T	902	0.78 (0.62–0.98)	0.0313	601	0.81 (0.62–1.05)	0.1168	301	0.71 (0.46–1.09)	0.1187
rs12204668	C/T	901	0.81 (0.64–1.02)	0.0678	601	0.82 (0.64–1.07)	0.1399	300	0.74 (0.43–1.25)	0.2586
rs1076733	A/G	901	1.18 (0.95–1.47)	0.1316	600	1.15 (0.89–1.48)	0.2836	301	1.28 (0.84–1.95)	0.2521
**rs7769547**	**A/G**	904	**0.70 (0.56**–**0.87)**	**0.0013**	603	**0.75 (0.58**–**0.97)**	**0.0304**	301	**0.58 (0.38**–**0.88)**	**0.0102**
rs2300613	A/G	902	0.72 (0.57–0.91)	0.0068	602	0.70 (0.53–0.92)	0.0104	300	0.79 (0.48–1.28)	0.3397
rs2268640	G/A	905	1.03 (0.83–1.30)	0.7665	603	0.98 (0.77–1.25)	0.8631	302	1.48 (0.78–2.82)	0.2324
rs2206942	T/C	905	0.91 (0.73–1.12)	0.3689	603	0.88 (0.69–1.13)	0.3084	302	1.00 (0.63–1.58)	0.9988
**rs10305512**	**A/G**	902	**0.53 (0.37**–**0.76)**	**0.0006**	602	**0.37 (0.20**–**0.66)**	**0.0009**	300	**0.66 (0.42**–**1.05)**	**0.0787**
rs10305514	T/G	906	0.93 (0.62–1.39)	0.7230	604	0.85 (0.51–1.43)	0.5365	302	1.07 (0.55–2.05)	0.8462
rs4714210	G/A	902	1.03 (0.82–1.29)	0.8197	601	1.06 (0.83–1.36)	0.6400	301	0.89 (0.54–1.48)	0.6586
rs4254984	C/T	903	0.98 (0.78–1.23)	0.8632	602	1.03 (0.80–1.32)	0.8420	301	0.80 (0.47–1.35)	0.4026
rs9968886	A/G	906	0.87 (0.67–1.14)	0.3187	604	0.85 (0.60–1.21)	0.3628	302	0.90 (0.60–1.37)	0.6369

Abbreviations: CI, confidence interval; *GLP1R*; glucagon-like peptide-1 receptor; OR, odds ratio; SNP, single-nucleotide polymorphism.

a Logistic regression controlling for ancestry; the effective number of independent marker loci (MeffLi) correction was used with a cut-off *P*-value of 0.0032. SNPs meeting this cut-off in the whole sample are in bold.

b *Post hoc* analysis using logistic regression; *P*-values were not adjusted.

**Table 5:** Measures from the IV-ASA experiment sorted by the rs6923761 genotype

	*168Gly/Gly (*N*=49)*	*168Ser/Gly (*N*=26)*	*168Ser/Ser (*N*=5)*	β*/OR*	P*-value*
Average BrAC (mg%)	34.39 (±28.99)	40.57 (±26.71)	57.26 (±31.49)	8.874	0.048
Peak BrAC (mg%)	53.37 (±37.26)	65.72 (±38.49)	83.59 (±40.33)	13.864	0.026
Number of button presses *ad lib*	11.39 (±7.85)	11.85 (±7.64)	15.60 (±8.85)	1.195	0.211
Total alcohol infused	33.02 (±23.50)	40.11 (±25.05)	47.11 (±25.05)	6.771	0.052
Binge ⩾80 mg%: yes (%)^a^	18 (36.73)	13 (50.00)	4 (80.00)	2.159	0.037^b^

Abbreviations: BrAC, breath alcohol concentration; OR, odds ratio.

a Data are presented as number of subjects reaching a BrAC of 80 mg% signifying a binge session.

b *P*-values are one-tailed and obtained by logistic regression and an additive genetic model. All analyses were controlled for age, body mass index, gender and ancestry.

All data are presented as mean (s.d.) unless specified otherwise. *P*-values are one-tailed and obtained by linear regression unless specified otherwise.

## Notes

EJ has received financial support from the Novo Nordisk Foundation. JAE has received financial support as consultant from Pfizer and Lundbeck. The other authors, PS, JY, MLS, BLS, ECC, RM, CAH, ME, MFL, HCB, DG, MH, VAR and LL report no financial relationships with commercial interests.
