# Supplementary material for: The glucagon-like peptide-1 receptor as a potential treatment target in alcohol use disorder: evidence from human genetic association studies and a mouse model of alcohol dependence
Source: Transl Psychiatry. 2015 Jun 16;5(6):e583–. doi: 10.1038/tp.2015.68 (PMC4490279; doi:10.1038/tp.2015.68)
Supplement: Supplementary Information [file tp201568x1.doc]

**SUPPLEMENTARY MATERIAL**

***Supplementary Methods***

***Haplotype analyses (Sudies 1 & 2)***

Haplotype blocks were constructed using Haploview (version 4.2) ^1^ and haplotype frequencies were estimated using the expectation-maximization (EM) algorithm and compared between cases and controls using the --hap-assoc command in PLINK (version 1.07, Shaun Purcell, http://pngu.mgh.harvard.edu/purcell/plink/).^2, 3^

***Supplementary Results***

*Haplotype analyses (Study 1)*

LD was investigated in control subjects of either ancestral group (Figure S1a and b). A haplotype block consisting of five SNPs in LD was constructed including two of the associated SNPs and two missense SNPs (rs6923761, Gly168Ser; rs1042044, Phe260Leu) reported to be functional in an *in vitro* study ^4^. The latter two, did not meet the MeffLi threshold for a significant p-value in this study but were both nominally associated with AUD (*p*=0.0047 and 0.0512, respectively) as was the fifth marker (rs7341356; p=0.0035) also included in the haplotype block. Haplotype frequencies were estimated using the EM-algorithm ^3^ and compared between cases and controls in both self-reported ancestral groups (Table S4 S7). The rs6923761 168Ser (A) allele was always present together with the other risk alleles, and this haplotype (ATGCC) was significantly more common among Caucasian AUD subjects when compared to Caucasian control subjects. In contrast, when the 168Gly (G) allele was present with the other risk alleles (GTGCC), haplotype frequencies did not differ between cases and controls. The haplotype consisting of the 260Phe (C allele in the GGAAC haplotype) as the only risk allele was also significantly more common among the AUD group. These results were not replicated in the African-American sample and a great discrepancy in frequencies for the associated haplotypes between this group and the Caucasian sample was observed (Table S4).

*Haplotype analysis (Study 2)*

We examined whether a haplotype analysis in the SAGE males would replicate the results from the LCTS population. The SNPs in this block were the same except for rs7766663, which was not genotyped in SAGE. We found a trend toward association for the haplotype comprising the 168Ser allele, with higher frequencies in alcohol dependent subjects compared to controls among Caucasians (Table S4, lower part). This was replicated among African-American subjects.

***Supplementary Discussion***

In addition to single marker associations, we analyzed a haplotype block including the Gly168Ser and Phe260Leu SNPs. The studied haplotype spans from transmembrane segment 1 to the intracellular loop 2 which is in close proximity to the transmembrane segment 4 previously reported to have an important role in the receptor’s ability to dimerize and thereby efficiently couple to its G-protein signaling cascade.^5^ Interestingly, the 168Ser allele was always on the same haplotype as the other identified risk alleles in this block. In Caucasians, the haplotype containing the 168Ser allele was more common among AUD subjects in the LCTS sample. This was replicated among male alcohol-dependent individuals in both the Caucasians and African-American subsamples of the SAGE population. Additionally, the haplotype block including the 260Phe as the only risk allele was significantly associated with AUD in the LCTS Caucasian sample. On the basis of these results we postulate that the 168Ser allele accounts for the primary AUD susceptibility originating from this locus, and drives the association, while the 260Phe risk allele has a greater impact when expressed in the absence of other risk alleles in the haplotype block.

**Supplemental References**

1. Barrett JC, Fry B, Maller J, Daly MJ. Haploview: Analysis and visualization of LD and haplotype maps. *Bioinformatics* 2005; **21**(2)**:** 263-265.

2. Purcell S, Neale B, Todd-Brown K, Thomas L, Ferreira MAR, Bender D*, et al*. PLINK: A Tool Set for Whole-Genome Association and Population-Based Linkage Analyses. *The American Journal of Human Genetics* 2007; **81**(3)**:** 559-575.

3. Excoffier L, Slatkin M. Maximum-likelihood estimation of molecular haplotype frequencies in a diploid population. *Mol Biol Evol* 1995; **12**(5)**:** 921-927.

4. Koole C, Wootten D, Simms J, Valant C, Miller LJ, Christopoulos A*, et al*. Polymorphism and Ligand Dependent Changes in Human Glucagon-Like Peptide-1 Receptor (GLP-1R) Function: Allosteric Rescue of Loss of Function Mutation. *Mol Pharmacol* 2011; **80**(3)**:** 486-497.

5. Harikumar KG, Wootten D, Pinon DI, Koole C, Ball AM, Furness SGB*, et al*. Glucagon-like peptide-1 receptor dimerization differentially regulates agonist signaling but does not affect small molecule allostery. *Proc Natl Acad Sci U S A* 2012; **109**(45)**:** 18607-18612.

**Supplemental Tables**

| Table S1 List of SNPs located in the *GLP1R* that were included on the Illumina OmniExpress BeadChip array. | | | | | | | | | | |
| --- | --- | --- | --- | --- | --- | --- | --- | --- | --- | --- |
|  |  |  |  |  | **Caucasian** | | | **African American** | | |
| **SNP no.^1^** | ***GLP1R* SNP** | **position** | **SNP type** | **minor allele/ major allele** | **HWE p-value controls** | **MAF controls** | **MAF AUD** | **HWE p-value controls** | **MAF controls** | **MAF AUD** |
| 1 | rs7738586 | 39007539 | 5' flanking region | A/C | 0.881 | 12.4% | 9.6% | 0.764 | 12.0% | 9.3% |
| 2 | rs9296274 | 39015117 | 5' flanking region | G/A | 0.247 | 12.1% | 10.8% | 0.027 | 26.9% | 20.6% |
| X | rs10305416 | 39015468 | 5' flanking region | A/G | 0.648 | 3.3% | 3.8% | 0.062 | 11.1% | 8.7% |
| 3 | rs2268657 | 39020541 | intron | C/T | 0.232 | 46.2% | 46.2% | 0.247 | 33.3% | 31.4% |
| 4 | rs3799707 | 39023509 | intron | T/G | 0.220 | 31.0% | 31.7% | 0.315 | 12.3% | 15.3% |
| X | rs10305432 | 39023629 | intron | C/T | 0.739 | 19.9% | 20.8% | 0.780 | 3.7% | 5.5% |
| X | rs2295006 | 39024224 | missense | A/G | - | 0.0% | 0.0% | - | 0.0% | 0.0% |
| X | rs10305438 | 39024447 | intron | A/G | 0.970 | 0.3% | 0.4% | 0.380 | 15.1% | 12.3% |
| 5 | rs10305439 | 39024715 | intron | A/C | 0.855 | 42.1% | 44.4% | 0.842 | 15.1% | 18.6% |
| 6 | rs2143734 | 39024755 | intron | G/A | 0.183 | 36.3% | 32.2% | 0.135 | 49.1% | 41.3% |
| X | rs10305441 | 39024838 | intron | A/G | 0.970 | 0.3% | 0.4% | - | 0.0% | 0.0% |
| X | rs9283907 | 39026702 | intron | A/G | 0.414 | 13.4% | 15.0% | 0.834 | 2.8% | 8.5% |
| 7 | rs2268650 | 39030400 | intron | A/G | 0.154 | 35.0% | 40.4% | 0.156 | 7.4% | 11.1% |
| 8 | rs910170 | 39032414 | intron | A/G | 0.921 | 47.0% | 44.0% | 0.688 | 50.0% | 43.5% |
| 9 | rs874900 | 39032536 | intron | G/A | 0.902 | 8.0% | 4.3% | 0.925 | 25.5% | 26.0% |
| X | rs6918287 | 39033601 | synonymous | A/G | 0.941 | 0.5% | 0.4% | - | 0.0% | 0.4% |
| 10 | rs6923761 | 39034071 | missense | A/G | 0.900 | 27.3% | 35.1% | 0.614 | 6.5% | 9.5% |
| 11 | rs7766663 | 39035781 | intron | T/G | 0.759 | 42.4% | 49.1% | 0.049 | 19.4% | 31.4% |
| X | rs12214482 | 39036184 | intron | G/A | 0.422 | 17.4% | 17.7% | 0.724 | 3.7% | 8.5% |
| 12 | rs7341356 | 39036344 | intron | G/A | 0.037 | 48.2% | 45.1% | 0.116 | 26.6% | 37.7% |
| 13 | rs2235868 | 39040653 | synonymous | C/A | 0.232 | 46.2% | 45.7% | 0.130 | 25.0% | 35.9% |
| X | rs10305475 | 39040700 | synonymous | G/A | - | 0.0% | 0.0% | 0.509 | 8.3% | 6.5% |
| 14 | rs1042044 | 39041501 | missense | A/C | 0.921 | 47.0% | 42.7% | 0.355 | 48.1% | 40.4% |
| 15 | rs932443 | 39042333 | intron | C/T | 0.899 | 36.4% | 31.9% | 0.657 | 40.7% | 32.8% |
| 16 | rs12204668 | 39043795 | intron | C/T | 0.931 | 35.2% | 39.7% | 0.239 | 21.7% | 26.7% |
| X | rs2268645 | 39043991 | intron | A/G | 0.996 | 7.4% | 7.0% | - | 0.0% | 2.0% |
| 17 | rs1076733 | 39045907 | intron | A/G | 0.897 | 42.9% | 46.2% | 0.458 | 54.6% | 48.6% |
| X | rs742761 | 39046654 | intron | T/C | 0.865 | 6.8% | 6.5% | 0.777 | 3.8% | 3.6% |
| 18 | rs7769547 | 39048756 | intron | A/G^3^ | 0.548 | 46.2% | 39.6% | 0.366 | 61.1% | 46.8% |
| 19 | rs2300613 | 39049204 | intron | A/G | 0.869 | 35.2% | 28.0% | 0.941 | 27.4% | 23.1% |
| 20 | rs2268640 | 39050383 | intron | G/A | 0.623 | 45.7% | 45.1% | 0.764 | 12.0% | 16.5% |
| 21 | rs2206942 | 39051438 | intron | T/C | 0.954 | 46.7% | 43.6% | 0.379 | 29.6% | 29.6% |
| 22 | rs10305512 | 39054112 | 3'UTR | A/G | 0.865 | 6.8% | 2.6% | 0.974 | 29.6% | 21.5% |
| 23 | rs10305514 | 39054137 | 3'UTR | T/G | 0.366 | 6.3% | 5.4% | 0.320 | 12.0% | 12.7% |
| X | rs10305518^2^ | 39055011 | 3'UTR | G/T | 0.366 | 6.3% | 5.4% | 0.320 | 12.0% | 12.7% |
| 24 | rs4714210 | 39055484 | 3'UTR | G/A | 0.925 | 39.0% | 40.5% | 0.957 | 23.1% | 21.3% |
| X | rs10305522 | 39055515 | 3'UTR | C/T | 0.569 | 9.5% | 11.1% | 0.946 | 0.9% | 1.8% |
| 25 | rs4254984 | 39059678 | 3' flanking region | C/T | 0.364 | 37.0% | 37.6% | 0.519 | 22.2% | 18.8% |
| 26 | rs9968886 | 39060477 | 3' flanking region | A/G | 0.751 | 15.8% | 13.8% | 0.653 | 39.8% | 42.3% |
| X | rs16891605 | 39071234 | 3' flanking region | C/T | 0.704 | 2.7% | 2.4% | 0.509 | 8.3% | 6.7% |

Hardy-Weinberg Equilibrium, HWE; Minor Allele Frequency, MAF; Alcohol Use Disorder, AUD; ^1^ X signifies SNPs that were removed from the analysis due to MAF < 5% (all SNPs past the HWE cut-off p-value of <0.01). ^2^ SNP is in complete LD (r^2^=1) with rs10305514 and was thus removed from the analysis. ^3^ For African American controls the G allele was the minor allele.

| Table S2 Genotype frequency comparison between cases and controls in Caucasian and African Americans separately. | | | | | | | | | | | |
| --- | --- | --- | --- | --- | --- | --- | --- | --- | --- | --- | --- |
|  |  |  |  | **Caucasian** | | | | **African Americans** | | | |
| **SNP no.** | **GLP1R SNP** | **minor allele/ major allele** |  | **dd** | **Dd** | **DD** | **p-value** | **dd** | **Dd** | **DD** | **p-value** |
| 1 | rs7738586 | A/C | Control | 3(1.6) | 39(21.4) | 140(76.9) | 0.360 | 1(1.9) | 11(20.4) | 42(77.8) | 0.415 |
|  |  |  | Case | 4(1.0) | 73(17.4) | 343(81.7) |  | 2(0.8) | 42(16.9) | 204(82.3) |  |
| 2 | rs9296274 | G/A | Control | 1(0.5) | 42(23.1) | 139(76.4) | 0.510 | 7(13.0) | 15(27.8) | 32(59.3) | 0.031 |
|  |  |  | Case | 5(1.2) | 81(19.3) | 334(79.5) |  | 9(3.6) | 84(33.9) | 155(62.5) |  |
| 3 | rs2268657 | C/T | Control | 35(19.1) | 99(54.1) | 49(26.8) | 0.753 | 4(7.4) | 28(51.9) | 22(40.7) | 0.541 |
|  |  |  | Case | 87(20.9) | 211(50.6) | 119(28.5) |  | 24(9.7) | 107(43.3) | 116(47.0) |  |
| 4 | rs3799707 | T/G | Control | 14(7.7) | 85(46.7) | 83(45.6) | 0.598 | 0(0) | 13(24.5) | 40(75.5) | 0.739 |
|  |  |  | Case | 42(10) | 182(43.4) | 195(46.5) |  | 2(0.8) | 72(29.0) | 174(70.2) |  |
| 5 | rs10305439 | A/C | Control | 33(18) | 88(48.1) | 62(33.9) | 0.493 | 1(1.9) | 14(26.4) | 38(71.7) | 0.795 |
|  |  |  | Case | 76(18.1) | 221(52.6) | 123(29.3) |  | 8(3.2) | 76(30.8) | 163(66.0) |  |
| 6 | rs2143734 | G/A | Control | 20(10.9) | 93(50.8) | 70(38.3) | 0.266 | 10(18.5) | 33(61.1) | 11(20.4) | 0.241 |
|  |  |  | Case | 41(9.8) | 188(44.9) | 190(45.3) |  | 33(13.4) | 138(55.9) | 76(30.8) |  |
| 7 | rs2268650 | A/G | Control | 18(9.8) | 92(50.3) | 73(39.9) | 0.151 | 1(1.9) | 6(11.1) | 47(87.0) | 0.273 |
|  |  |  | Case | 63(15.1) | 211(50.6) | 143(34.3) |  | 3(1.2) | 49(19.8) | 196(79.0) |  |
| 8 | rs910170 | A/G | Control | 41(22.3) | 91(49.5) | 52(28.3) | 0.593 | 14(25.9) | 26(48.1) | 14(25.9) | 0.357 |
|  |  |  | Case | 79(18.9) | 211(50.4) | 129(30.8) |  | 43(17.4) | 129(52.2) | 75(30.4) |  |
| 9 | rs874900 | G/A | Control | 1(0.6) | 26(14.9) | 148(84.6) | 0.017 | 3(6.1) | 19(38.8) | 27(55.1) | 0.941 |
|  |  |  | Case | 2(0.5) | 29(7.5) | 355(92.0) |  | 19(7.9) | 87(36.3) | 134(55.8) |  |
| 10 | rs6923761 | A/G | Control | 14(7.7) | 72(39.3) | 97(53.0) | 0.027 | 0(0) | 7(13.0) | 47(87.0) | 0.532 |
|  |  |  | Case | 49(11.7) | 196(46.8) | 174(41.5) |  | 1(0.4) | 45(18.1) | 202(81.5) |  |
| 11 | rs7766663 | T/G | Control | 30(17.4) | 86(50.0) | 56(32.6) | 0.111 | 4(8.2) | 11(22.4) | 34(69.4) | 0.006 |
|  |  |  | Case | 91(23.2) | 204(51.9) | 98(24.9) |  | 20(8.8) | 103(45.2) | 105(46.1) |  |
| 12 | rs7341356 | G/A | Control | 39(22.8) | 99(57.9) | 33(19.3) | 0.069 | 5(10.6) | 15(31.9) | 27(57.4) | 0.025 |
|  |  |  | Case | 72(18.1) | 214(53.9) | 111(28.0) |  | 28(12.1) | 119(51.3) | 85(36.6) |  |
| 13 | rs2235868 | C/A | Control | 49(26.8) | 99(54.1) | 35(19.1) | 0.025 | 5(9.3) | 17(31.5) | 32(59.3) | 0.040 |
|  |  |  | Case | 78(18.6) | 227(54.2) | 114(27.2) |  | 29(11.7) | 120(48.4) | 99(39.9) |  |
| 14 | rs1042044 | A/C | Control | 41(22.3) | 91(49.5) | 52(28.3) | 0.285 | 14(25.9) | 24(44.4) | 16(29.6) | 0.230 |
|  |  |  | Case | 71(16.9) | 217(51.7) | 132(31.4) |  | 39(15.9) | 121(49.2) | 86(35.0) |  |
| 15 | rs932443 | C/T | Control | 24(13.0) | 86(46.7) | 74(40.2) | 0.214 | 10(18.5) | 24(44.4) | 20(37.0) | 0.212 |
|  |  |  | Case | 36(8.6) | 194(46.5) | 187(44.8) |  | 26(10.5) | 110(44.5) | 111(44.9) |  |
| 16 | rs12204668 | C/T | Control | 23(12.6) | 83(45.4) | 77(42.1) | 0.313 | 1(1.9) | 21(39.6) | 31(58.5) | 0.551 |
|  |  |  | Case | 63(15.1) | 206(49.3) | 149(35.6) |  | 13(5.3) | 106(42.9) | 128(51.8) |  |
| 17 | rs1076733 | A/G | Control | 33(18.1) | 90(49.5) | 59(32.4) | 0.563 | 15(27.8) | 29(53.7) | 10(18.5) | 0.462 |
|  |  |  | Case | 86(20.6) | 214(51.2) | 118(28.2) |  | 58(23.5) | 124(50.2) | 65(26.3) |  |
| 18 | rs7769547 | A/G | Control | 37(20.2) | 95(51.9) | 51(27.9) | 0.090 | 22(40.7) | 22(40.7) | 10(18.5) | 0.027 |
|  |  |  | Case | 60(14.3) | 213(50.7) | 147(35.0) |  | 57(23.1) | 117(47.4) | 73(29.6) |  |
| 19 | rs2300613 | A/G | Control | 22(12.1) | 84(46.2) | 76(41.8) | 0.021 | 4(7.5) | 21(39.6) | 28(52.8) | 0.484 |
|  |  |  | Case | 25(6.0) | 185(44.0) | 210(50.0) |  | 11(4.5) | 92(37.2) | 144(58.3) |  |
| 20 | rs2268640 | G/A | Control | 40(21.7) | 88(47.8) | 56(30.4) | 0.979 | 1(1.9) | 11(20.4) | 42(77.8) | 0.293 |
|  |  |  | Case | 88(21.0) | 202(48.2) | 129(30.8) |  | 4(1.6) | 74(29.8) | 170(68.5) |  |
| 21 | rs2206942 | T/C | Control | 40(21.7) | 92(50.0) | 52(28.3) | 0.587 | 6(11.1) | 20(37.0) | 28(51.9) | 0.571 |
|  |  |  | Case | 81(19.3) | 203(48.4) | 135(32.2) |  | 20(8.1) | 107(43.1) | 121(48.8) |  |
| 22 | rs10305512 | A/G | Control | 1(0.5) | 23(12.6) | 159(86.9) | 0.001 | 5(9.3) | 22(40.7) | 27(50.0) | 0.170 |
|  |  |  | Case | 0(0) | 22(5.3) | 397(94.7) |  | 13(5.3) | 80(32.5) | 153(62.2) |  |
| 23 | rs10305514 | T/G | Control | 0(0) | 23(12.5) | 161(87.5) | 0.412 | 0(0) | 13(24.1) | 41(75.9) | 0.923 |
|  |  |  | Case | 2(0.5) | 41(9.8) | 377(89.8) |  | 3(1.2) | 57(23.0) | 188(75.8) |  |
| 24 | rs4714210 | G/A | Control | 28(15.4) | 86(47.3) | 68(37.4) | 0.907 | 3(5.6) | 19(35.2) | 32(59.3) | 0.740 |
|  |  |  | Case | 69(16.5) | 201(48.0) | 149(35.6) |  | 9(3.6) | 87(35.2) | 151(61.1) |  |
| 25 | rs4254984 | C/T | Control | 28(15.2) | 80(43.5) | 76(41.3) | 0.530 | 2(3.7) | 20(37.0) | 32(59.3) | 0.628 |
|  |  |  | Case | 56(13.4) | 202(48.3) | 160(38.3) |  | 6(2.4) | 81(32.8) | 160(64.8) |  |
| 26 | rs9968886 | A/G | Control | 4(2.2) | 50(27.2) | 130(70.7) | 0.564 | 8(14.8) | 27(50.0) | 19(35.2) | 0.767 |
|  |  |  | Case | 5(1.2) | 106(25.2) | 309(73.6) |  | 48(19.4) | 114(46.0) | 86(34.7) |  |

Minor genotype (dd), heterozygote genotype (Dd), major genotype (DD). Data presented as N(%). P-values obtained by Fishers exact test.

| Table S3 Case-control analyses in the LCTS cohort in females and males separately. | | | | | | | |
| --- | --- | --- | --- | --- | --- | --- | --- |
|  |  | **Females** | | | **Males** | | |
| **GLP1R SNP** | **minor allele/ major allele** | **N** | **OR (95% CI)** | **p-value** | **N** | **OR (95% CI)** | **p-value** |
| rs6923761 | A/G | 296 | 1.15(0.75-1.77) | 0.5304 | 608 | 1.66(1.19-2.32) | 0.0031 |
| rs7766663 | T/G | 276 | 1.43(0.99-2.08) | 0.0581 | 566 | 1.40(1.04-1.89) | 0.0282 |
| rs2235868 | C/A | 296 | 1.37(0.96-1.96) | 0.0781 | 608 | 1.55(1.15-2.09) | 0.0042 |
| rs1042044 | A/C | 297 | 0.84(0.59-1.18) | 0.3091 | 607 | 0.79(0.60-1.05) | 0.1047 |
| rs7769547 | A/G | 296 | 0.67(0.47-0.96) | 0.0275 | 608 | 0.72(0.55-0.96) | 0.0237 |
| rs10305512 | A/G | 295 | 0.4(0.22-0.74) | 0.0035 | 607 | 0.65(0.41-1.03) | 0.0636 |

Statistics obtained using logistic regression controlling for self-reported ancestry.

| Table S4 Haplotype frequencies in controls and individuals with alcohol use disorder (AUD) or alcohol dependence (AD) in the LCTS and SAGE studies, respectively. | | | | | | | | | | |
| --- | --- | --- | --- | --- | --- | --- | --- | --- | --- | --- |
|  | **Caucasian** | | | | | **African-American** | | | | |
| **Haplotype** | **Controls** | **AUD/AD** | **CHISQ** | **df** | **p-value** | **Controls** | **AUD/AD** | **CHISQ** | **df** | **p-value** |
| *LCTS study* |  |  |  |  |  |  |  |  |  |  |
| G G A A A | 47.5% | 43.2% | 1.88 | 1 | 0.1707 | 49.1% | 40.5% | 2.65 | 1 | 0.1038 |
| **A T G C C** | 27.3% | 35.5% | 7.56 | 1 | 0.0060 | 6.6% | 9.5% | 0.89 | 1 | 0.3445 |
| G **T G C C** | 16.7% | 15.6% | 0.25 | 1 | 0.6194 | 16.7% | 24.2% | 2.82 | 1 | 0.0929 |
| G G A A **C** | 6.6% | 2.9% | 9.13 | 1 | 0.0025 | 26.4% | 23.8% | 0.33 | 1 | 0.5685 |
| G G **G C** **C** | 1.8% | 2.8% | 1.08 | 1 | 0.2998 | 1.3% | 2.0% | 0.27 | 1 | 0.6027 |
|  |  |  |  |  |  |  |  |  |  |  |
| *SAGE study* |  |  |  |  |  |  |  |  |  |  |
| G A A A | 46.6% | 43.0% | 2.84 | 1 | 0.0920 | 38.6% | 42.5% | 1.37 | 1 | 0.2412 |
| **A G C C** | 29.3% | 32.6% | 2.64 | 1 | 0.0521* | 4.5% | 8.3% | 5.03 | 1 | 0.0125* |
| G **G C C** | 18.1% | 19.5% | 0.65 | 1 | 0.4214 | 31.7% | 25.9% | 3.72 | 1 | 0.0538 |
| G A A **C** | 4.7% | 4.1% | 0.59 | 1 | 0.2218* | 25.2% | 23.3% | 0.48 | 1 | 0.2439* |
| G A C C | 1.2% | 0.9% | 0.59 | 1 | 0.4430 | 38.6% | 42.5% | 1.37 | 1 | 0.2412 |

Haplotype block consist of SNP 10-14 in Table S4 (rs6923761, rs7766663, rs734135, rs2235868 and rs1042044) for the LCTS study and 10, 12-14 for the SAGE study. Only male subjects from the SAGE study were included. Alleles nominally associated with AUD in the single marker analysis are bolded. Frequencies estimated using the EM-algorithm. Only haplotypes with a frequency ≥1% are included. One-sided p-values are marked with an asterix.

**Supplemental figures**

**Figure S1**

Linkage disequilibrium structure of *GLP1R* in Caucasian controls (A) and African-American controls (B). SNP numbers correspond to the numbers seen in Table S4. Shading of diamonds indicate the degree of correlation (D'; larger D' the darker the shading), values in diamonds are r^2^. The haplotype block was constructed on the basis of SNPs nominally associated with alcohol use disorder in Table 1.

**S1A
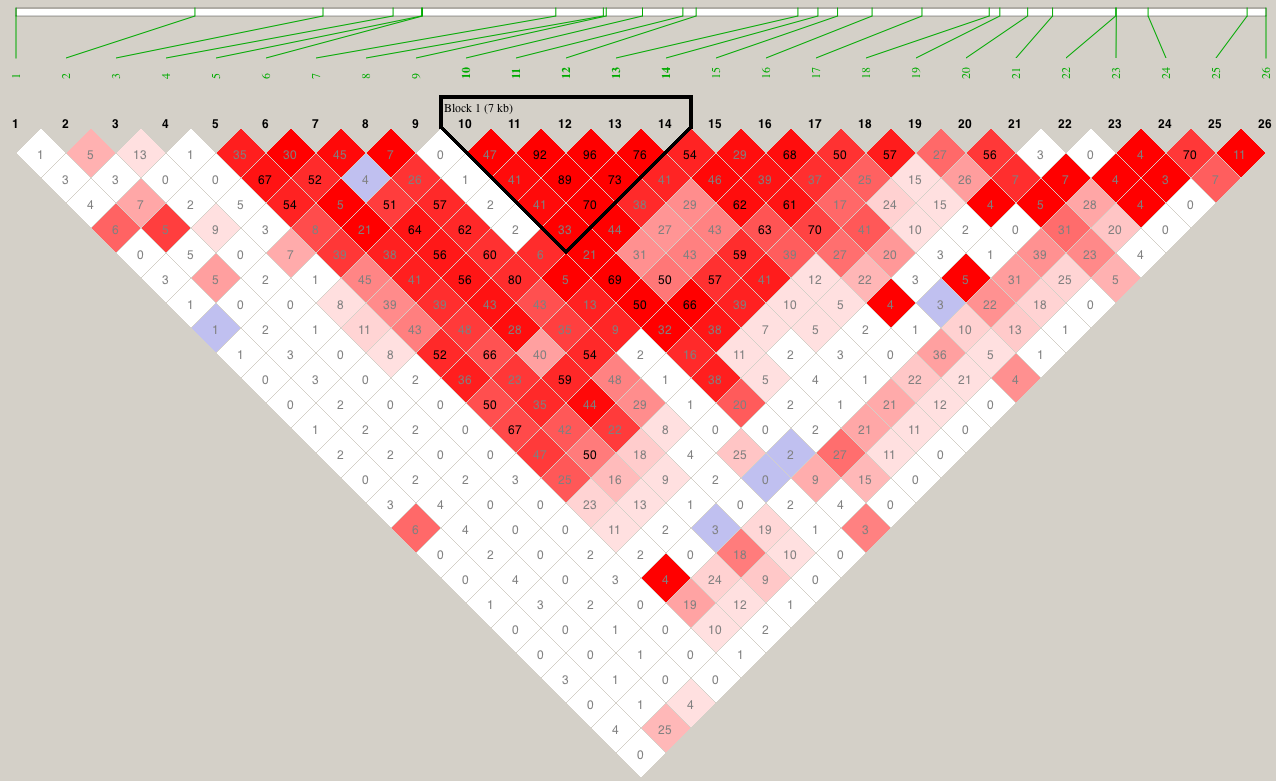
**

**S1B**

**
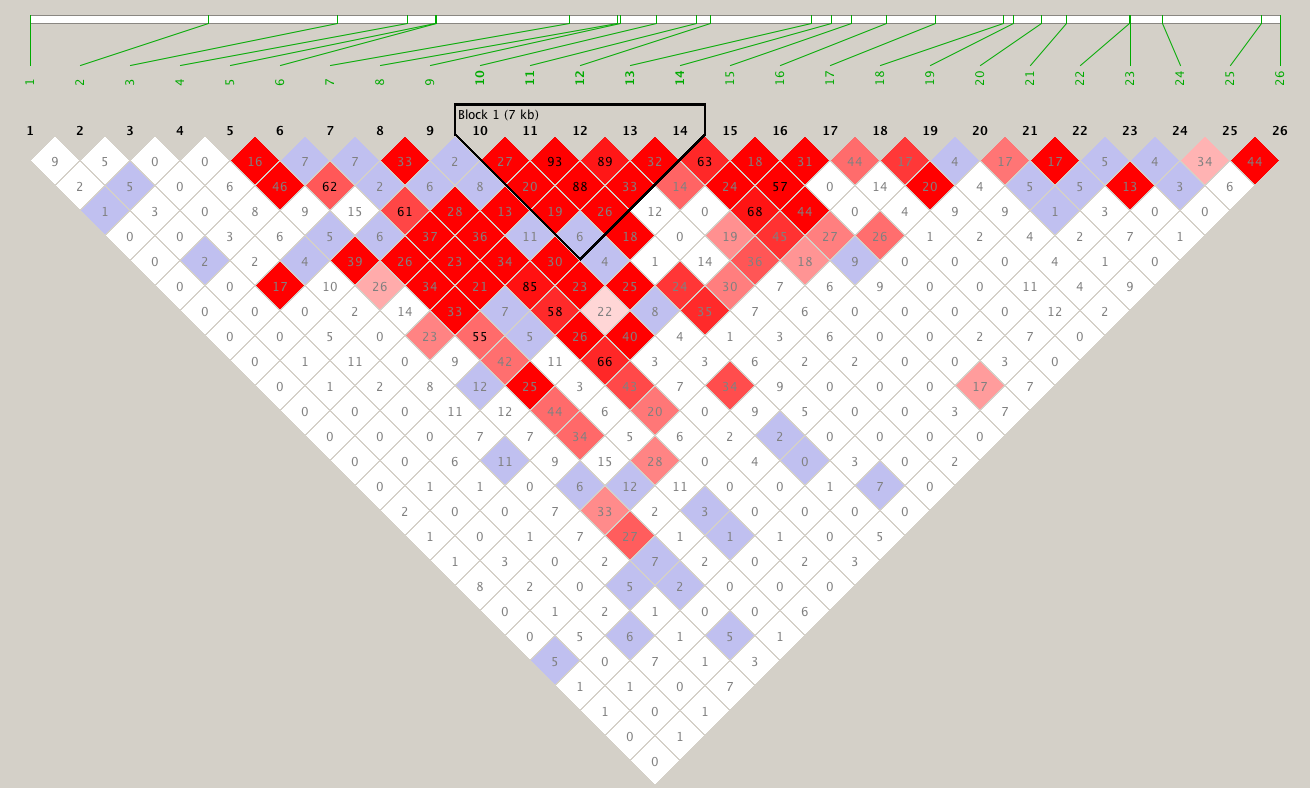
**

**Figure S2. Schematic of Study Design for Study 5 (mouse model of dependence and relapse drinking).** After stable baseline free-choice drinking was established, adult male C57BL/6J mice were exposed to repeated weekly cycles of chronic intermittent ethanol (CIE) or air inhalation exposure alternated with weekly test cycles of voluntary ethanol drinking. Mice received vehicle (saline) injections prior to Baseline and the first four test cycle drinking sessions (Tests Cycle 1-4) and then different doses of AC3174 prior to Test Cycles 5, 6, and 7. All mice again received vehicle injections for drug-washout testing during Test Cycles 8 and 9. See text for procedural details.

**S2**
